# Infrapatellar fat pad adipose tissue-derived macrophages display a predominant CD11c+CD206+ phenotype and express genotypes attributable to key features of OA pathogenesis

**DOI:** 10.3389/fimmu.2024.1326953

**Published:** 2024-02-01

**Authors:** Patchanika Hengtrakool, Nitigorn Leearamwat, Panjana Sengprasert, Jutamas Wongphoom, Thiamjit Chaichana, Mana Taweevisit, Srihatach Ngarmukos, Aree Tanavalee, Tanapat Palaga, Rangsima Reantragoon

**Affiliations:** ^1^ Medical Microbiology Interdisciplinary Program, Graduate School, Chulalongkorn University, Bangkok, Thailand; ^2^ Immunology Division, Department of Microbiology, Faculty of Medicine, Chulalongkorn University, Bangkok, Thailand; ^3^ Department of Pathology, King Chulalongkorn Memorial Hospital, Bangkok, Thailand; ^4^ Department of Pathology, Faculty of Medicine, Chulalongkorn University, Bangkok, Thailand; ^5^ Department of Orthopedics, Faculty of Medicine, Chulalongkorn University, Bangkok, Thailand; ^6^ Biologics for Knee Osteoarthritis Research Unit, Faculty of Medicine, Chulalongkorn University, Bangkok, Thailand; ^7^ Department of Microbiology, Faculty of Science, Chulalongkorn University, Bangkok, Thailand; ^8^ Center of Excellence in Immunology and Immune-Mediated Diseases, Faculty of Medicine, Chulalongkorn University, Bangkok, Thailand; ^9^ Center of Excellence in Skeletal Disorders and Enzyme Reaction Mechanism, Faculty of Dentistry, Chulalongkorn University, Bangkok, Thailand

**Keywords:** osteoarthritis, macrophage, macrophage polarization, synovial tissue, infrapatellar fat pad

## Abstract

**Objectives:**

In knee osteoarthritis (OA), macrophages are the most predominant immune cells that infiltrate synovial tissues and infrapatellar fat pads (IPFPs). Both M1 and M2 macrophages have been described, but their role in OA has not been fully investigated. Therefore, we investigated macrophage subpopulations in IPFPs and synovial tissues of knee OA patients and their correlation with disease severity, examined their transcriptomics, and tested for factors that influenced their polarization.

**Methods:**

Synovial tissues and IPFPs were obtained from knee OA patients undergoing total knee arthroplasty. Macrophages isolated from these joint tissues were characterized via flow cytometry. Transcriptomic profiling of each macrophage subpopulations was performed using NanoString technology. Peripheral blood monocyte-derived macrophages (MDMs) were treated with synovial fluid and synovial tissue- and IPFP-conditioned media. Synovial fluid-treated MDMs were treated with platelet-rich plasma (PRP) and its effects on macrophage polarization were observed.

**Results:**

Our findings show that CD11c+CD206+ macrophages were predominant in IPFPs and synovial tissues compared to other macrophage subpopulations (CD11c+CD206-, CD11c-CD206+, and CD11c-CD206- macrophages) of knee OA patients. The abundance of macrophages in IPFPs reflected those in synovial tissues but did not correlate with disease severity as determined from Mankin scoring of cartilage destruction. Our transcriptomics data demonstrated highly expressed genes that were related to OA pathogenesis in CD11c+CD206+ macrophages than CD11c+CD206-, CD11c-CD206+, and CD11c-CD206- macrophages. In addition, MDMs treated with synovial fluid, synovial tissue-conditioned media, or IPFP-conditioned media resulted in different polarization profiles of MDMs. IPFP-conditioned media induced increases in CD86+CD206+ MDMs, whereas synovial tissue-conditioned media induced increases in CD86+CD206- MDMs. Synovial fluid treatment (at 1:8 dilution) induced a very subtle polarization in each macrophage subpopulation. PRP was able to shift macrophage subpopulations and partially reverse the profiles of synovial fluid-treated MDMs.

**Conclusion:**

Our study provides an insight on the phenotypes and genotypes of macrophages found in IPFPs and synovial tissues of knee OA patients. We also show that the microenvironment plays a role in driving macrophages to polarize differently and shifting macrophage profiles can be reversed by PRP.

## 1 Introduction

Osteoarthritis (OA) is the most common degenerative joint disease affecting the middle-aged and elderly worldwide (1). Evidence of immune responses involved in OA pathogenesis include low-grade inflammation, immune cell infiltration in the joint tissues, autoantibodies against self-cartilage components, cytokines released from immune cells in the joint, and complement activation in the synovium (2–4). Synovial tissues and infrapatellar fat pads (IPFPs) are potential sources of proinflammatory cytokines and growth factors associated with joint inflammation and pain in knee OA patients (5, 6). The inflamed synovium and IPFPs of OA patients are infiltrated with immune cells (7, 8).

Macrophages are a predominant component of mononuclear cell infiltration in synovial tissues and IPFPs and are highly activated in OA (7–9). Previous research has revealed the correlation between synovial macrophages with several OA pathologies (10, 11). A high percentage of synovial macrophages was detected in the synovial tissues of OA patients with moderate-grade synovitis, suggesting the association between macrophages with synovitis (12).

Macrophages are generally divided into M1 and M2 macrophages based on their polarization (13). M1 macrophages are associated with inflammation and matrix metallopeptidase (MMP) production (14), while M2 macrophages initiate the repair of joint tissue injury and articular cartilage damage (15, 16). In some pathological conditions, a mixed M1/M2 macrophage population was also described (17, 18). In OA, polarization of resident macrophages within the joint-surrounding tissues has not been well characterized. Therefore, we aimed to investigate the different macrophage subpopulations and their polarization in knee OA.

## 2 Materials and methods

### 2.1 Clinical samples

Synovial tissues, IPFPs, articular cartilage, and synovial fluid (SF) were collected from knee OA patients who underwent total knee arthroplasty at King Chulalongkorn Memorial Hospital. Ethical approval was obtained from the ethical committee of the Institutional Review Board (IRB) at the Faculty of Medicine, Chulalongkorn University, Bangkok, Thailand (IRB no. 0734/65) and with the Helsinki Declaration of 1975, as revised in 2000. All patients provided written informed consent.

### 2.2 Macrophage isolation from synovial tissues and IPFPs

The isolation of macrophages was adapted from Cassetta et al. (19). Synovial tissues and IPFPs were cut into small pieces in petri dishes and transferred to serum-free phosphate-buffered saline (PBS). Liberase DL (28 U/mL) (Roche, Basel, Switzerland), Liberase TL (14 U/mL) (Roche, Basel, Switzerland), and DNase I (15 mg/mL) (Thermo Scientific, Waltham, Massachusetts, US) were added, incubated for 1 h at 37°C, and vortexed at 58*g*. Then, cells were subsequently filtered using a 100-µm cell strainer and centrifuged at 524*g* for 10 min at 4°C. Cell pellets were harvested, washed once with RF10 medium (RPMI containing 10% fetal bovine serum (FBS), 20% non-essential amino acids, 0.6% L-glutamine, 2% HEPES, 0.007% β-mercaptoethanol, and 1 mM of sodium pyruvate) (all from Gibco, Waltham, Massachusetts, US), resuspended in freezing medium (10% DMSO in FBS), and stored in liquid nitrogen.

### 2.3 Macrophage phenotype determination and macrophage sorting

Macrophages were seeded into 96-well V-shaped-bottom plates (2 × 10^5^ cells/well) (Thermo Scientific, Waltham, Massachusetts, US) and washed with FACS buffer (2% FBS in PBS). Zombie aqua (Biolegend, San Diego, California, US) was used to exclude dead cells at a dilution of 1:1,000. For cell surface marker staining, cells were incubated in a final volume of 50 μL at 4°C for 45 min in the dark with the following antibodies: anti-human CD3-FITC (HIT3a), anti-human CD11b-APC/Cyanine7 (ICRF44), anti-human CD11c-PerCP/Cyanine 5.5 (Bu15), anti-human CD14-PE (63D3), anti-human CD206-PE/Dazzle™ 594 (15–2), and anti-human HLA-DR-Alexa Fluor^®^ 647 (L243) (all from Biolegend, San Diego, California, US). After washing twice with FACS buffer, the cells were treated with a fixation buffer (2% FBS and 1% formaldehyde in PBS) at 4°C for 1 h and subsequently permeabilized and incubated with 1% saponin (Sigma Aldrich, St. Louis, Missouri, US) in PBS containing anti-human CD68-PE/Cyanine7 (Y1/82A) (Biolegend, San Diego, California, US) in FACS buffer at 4°C for 1 h in the dark. The cells were acquired on CytoFLEX (Beckman Coulter, Brea, California, US), and cell sorting was performed on BD LSR II (BD Biosciences, Franklin Lakes, New Jersey, US). The results were analyzed with Flowjo software (Treestar).

### 2.4 Cartilage histopathology assessment

Cartilage harvesting and processing protocols were adapted from Pauli et al. (20). Briefly, articular cartilage samples were harvested in 10% neutral buffered formalin for 72 h, decalcified with 10% formic acid for another 48 to 72 h depending on the cartilage size, and cut into smaller tissue blocks. After dehydrating in a series of increasing concentrations of alcohol solution, the tissue blocks were embedded in paraffin and cut into 3-µm sections. Each section was stained with Safranin O and Fast Green for proteoglycan content and bone staining. The cartilage sections were scored for severity using the Mankin scoring system (**Supplementary Table S1**) (21). In this study, score ranging was divided into three grades: mild (0–4), moderate (5–9), and severe (10–14).

### 2.5 NanoString gene expression analysis of IPFP-isolated macrophages

Macrophages were isolated from the IPFPs of 14 knee OA patients and pooled into one sample. In total, three pooled samples were generated. CD11c-CD206-, CD11c+CD206-, CD11c-CD206+, and CD11c+CD206+ macrophages were sorted on BD LSR II (BD Biosciences, Franklin Lakes, New Jersey, US) from the pooled samples. The sorted macrophages were collected, and total RNA was extracted using the RNeasy Mini Kit (QIAGEN) according to the manufacturer’s instructions. Total RNA concentration was measured by using Nanodrop and Qubit fluorometers (Invitrogen).

Total RNA was converted to cDNA and amplified with a multiplex low-input primer pool using the nCounter Low RNA Input Kit (NanoString Technologies). The amplified products were quantified by using Nanodrop, and 10 mg of total cDNA was subsequently hybridized to a reporter and capture probe set of the nCounter Myeloid Innate Immunity Gene Expression Panel (NanoString Technologies) at 65°C for 18 h using a thermal cycler (Biorad). The hybridized samples were loaded on to the nCounter cartridge, and post-hybridization processing was carried out on a fully automated nCounter Prep station. The bound probe–gene target complexes were immobilized on the cartridge, and signals were subsequently read using the nCounter MAX/FLEX digital analyzer. Data were analyzed using the nSolver analysis software (NanoString Technologies). The positive and negative controls included in the probe sets were used for setting background thresholds and normalizing samples for differences in hybridization or sample inputs, respectively.

Raw data from the digital analyzer were evaluated for quality and normalized to internal positive and negative controls and a geometric mean of 38 housekeeping genes. The expression values of genes were calculated as log_2_ fold change from the mean of internal negative control and were considered as upregulated and downregulated when the log_2_ fold change expression value was greater than 0.5 and lower than -0.5, respectively. Venn diagrams were generated among the four subpopulations of macrophages using InteractiVenn software (online on http://www.interactivenn.net) (22). Heatmaps were generated based on log_2_ fold change values of gene expression using GraphPad Prism (version 8).

### 2.6 Determination of cytokine expression via cytometric bead array

Macrophages were isolated from three pooled samples (two individuals with knee OA combined into one sample). CD11c-CD206-, CD11c+CD206-, CD11c-CD206+, and CD11c+CD206+ macrophages were sorted on BD LSR II (BD Biosciences, Franklin Lakes, New Jersey, US) from the pooled samples. Each of the four macrophage subpopulations was cultured for 24 h in RF10 medium, and supernatant was collected. A customized human LEGENDplex™ panel kit (BioLegend, San Diego, California, US) was used to determine the concentrations of IL-17A, IL-1β, IL-13, IL-4, IL-10, TNF-α, IL-6, and IL-1RA as per the manufacturer’s instructions. The supernatant of each of the four macrophage subpopulations was mixed with assay buffer at a ratio of 1:1, incubated with antibody-coated beads, and oscillated at 84*g* on a plate shaker for 2 h at room temperature. Streptavidin–phycoerythrin was added and incubated with oscillation for 30 min at room temperature. The beads were then washed twice with wash buffer and centrifuged at 1,000*g* for 5 min. Samples were acquired on CytoFLEX (Beckman Coulter, Brea, California, US) and analyzed with LEGENDplex™ software (BioLegend, San Diego, California, US).

### 2.7 Enzyme-linked immunosorbent assay for the detection of MMP-9 and MMP-13

The supernatants of 24-h cultures of CD11c-CD206-, CD11c+CD206-, CD11c-CD206+, and CD11c+CD206+ macrophages were used to determine the presence of MMP-9 and MMP-13 production using DuoSet™ ELISA Kits (R&D system). Protocols were performed as per the manufacturer’s instructions. Briefly, 96-well microplates were coated with 1 or 4 μg/mL of captured antibodies specific to MMP-9 or MMP-13, respectively, in 1× PBS and incubated overnight at room temperature. The plates were washed with wash buffer (0.05% Tween20 in 1× PBS) three times and incubated with blocking buffer (1% BSA in 1× PBS) for 1 h at room temperature. The plates were washed with wash buffer three times and incubated with a standard or macrophage culture supernatant for 2 h at room temperature. After washing three times, detecting antibodies specific to MMP-9 or MMP-13 were diluted in a reagent diluent (1% BSA in 1× PBS), added to each well, and incubated for 2 h at room temperature. The plates were washed again three times and incubated with a substrate solution (1:1 mixture of H_2_O_2_ and tetramethylbenzidine) for 20 min at room temperature in the dark. The reactions were stopped by the addition of a stop solution (2 N H_2_SO_4_). Optical density (OD) values were determined at dual wavelengths of 450 and 570 nm using a microplate reader (Thermo Scientific, Waltham, Massachusetts, US). The OD values were plotted against the concentration of the standard samples to create a standard curve. The equation of the line from the standard curve was used to calculate the concentration of cytokines in the samples.

### 2.8 Determination of nuclear factor of activated T cells, cytoplasmic 1 and tartrate-resistant acid phosphatase expression in macrophages

Macrophages were seeded into 96-well V-shaped-bottom plates (2 × 10^5^ cells/well) (Thermo Scientific, Waltham, Massachusetts, US), washed with FACS buffer, and centrifuged at 524*g* for 5 min at 4°C. Zombie aqua (Biolegend, San Diego, California, US) was used to exclude dead cells at a dilution of 1:1,000. For cell surface marker staining, cells were incubated at 4°C for 45 min in the dark in a final volume of 50 μL with the following antibodies: anti-human CD11b-APC/Cyanine7 (ICRF44), anti-human CD11c-PerCP/Cyanine 5.5 (Bu15), anti-human CD14-PE (63D3), anti-human CD206-PE/Dazzle™ 594 (15–2), anti-human HLA-DR-Alexa Fluor^®^ 647 (L243), and anti-human tartrate-resistant acid phosphatase (TRAP)-BV421™ (23–30) (all from Biolegend, San Diego, California, US). After washing twice with FACS buffer, the cells were treated with a fixation buffer at 4°C for 1 h and subsequently permeabilized and incubated with 1% saponin (Sigma Aldrich, St. Louis, Missouri, US) in PBS containing anti-human nuclear factor of activated T cells, cytoplasmic 1 (NFATC1)-AF488 (7A6), and anti-human CD68-PE/Cyanine7 (Y1/82A) (Biolegend, San Diego, California, US) in FACS buffer at 4°C for 1 h in the dark. The cells were acquired on CytoFLEX (Beckman Coulter, Brea, California, US), and the results were analyzed with Flowjo software (Treestar).

### 2.9 Peripheral blood monocyte-derived macrophage differentiation and polarization

Blood samples were collected in EDTA tubes, transferred to 10 mL of RF10 medium, and mixed gently. Mixed blood was layered onto Ficoll–Paque reagent (ratio of blood/medium/Ficoll–Paque reagent = 1:1:1) and centrifuged at 524*g* at room temperature for 30 min without deceleration. Mononuclear cells were gently transferred into 20 mL of RF10 medium and centrifuged at 524*g* at 4°C for 10 min. The supernatant was discarded, and the cells were resuspended at 2 × 10^6^ cells/mL in RF10 medium and cultured at 37°C in 5% v/v of CO_2_ for 24 h to allow for monocyte adhesion. After 24 h, non-adherent cells were removed, and the remaining adherent monocytes were washed with 1× PBS and harvested. The monocytes were seeded at 10^4^ cells/mL on 24-well plates and cultured in RF10 medium containing 50 ng/mL of macrophage colony-stimulating factor (M-CSF) (PeproTech) for 7 days in 5% v/v of CO_2_ at 37°C. For 7 days, the culture medium was replaced with RF10 medium supplemented with 50 ng/mL of M-CSF every 3 days. After 7 days, monocyte-derived macrophages (MDMs) were washed with 1× PBS, rested in RF10 medium, and stimulated with either 25 ng/mL of lipopolysaccharide (LPS) (Sigma Aldrich, St. Louis, Missouri, US) and 25 ng/mL of IFN-γ (R&D Systems) or 25 ng/mL of IL-4 (PeproTech) and 25 ng/mL of IL-13 (PeproTech) in RF10 medium for 48 h to induce M1 and M2 macrophage polarization, respectively.

### 2.10 IPFP- and synovial tissue-conditioned medium preparation

Synovial tissues and IPFPs were washed with 1× PBS twice, cut into small pieces in petri dishes, and cultured at a concentration of 300 mg/mL (w/v) in RF10 medium. After 3 h, the RF10 medium was replaced, and the tissues were cultured for another 24 h. Supernatant was collected and centrifuged at 524*g* for 5 min and stored at -80°C until use.

### 2.11 Determination of synovial fluid and joint tissue-conditioned medium-induced macrophage polarization

MDMs (10^4^ cells/well) were incubated with synovial fluid (1:2, 1:4, and 1:8 dilution) in culture medium or 100 mg/mL (w/v) of joint tissue cultured–conditioned medium for 48 h in 5% v/v of CO_2_ at 37°C. The treated MDMs were transferred into 96-well V-shaped-bottom plates (10^4^ cells/well), washed with FACS buffer, and centrifuged at 524*g* for 5 min at 4°C. Zombie aqua (Biolegend, San Diego, California, US) was used to exclude dead cells. For cell surface marker staining, the cells were incubated at 4°C for 45 min in the dark in a final volume of 50 μL with the following antibodies; anti-human CD11b-APC/Cyanine7 (ICRF44), anti-human CD14-PE (63D3), anti-human CD86-APC (IT2.2), and anti-human CD206-PE/Dazzle™ 594 (15–2) (all from Biolegend, San Diego, California, US). After washing twice with FACS buffer, the cells were fixed with a fixation buffer (2% FBS and 1% formaldehyde in PBS) at 4°C for 1 h and incubated with 1% saponin (Sigma Aldrich, St. Louis, Missouri, US) in PBS containing anti-human CD68-PE/Cyanine7 (Y1/82A) (Biolegend, San Diego, California, US) in FACS buffer at 4°C for 1 h in the dark. FACS analysis was performed on CytoFLEX (Beckman Coulter, Brea, California, US). The results were analyzed with Flowjo software (Treestar).

### 2.12 Platelet-rich plasma preparation

The platelet-rich plasma (PRP) preparation protocols were adapted from Perez AG et al. and Ngarmukos S et al. (31, 32). Peripheral blood was collected in anticoagulant citrate dextrose solution (Vacuette, Greiner Bio-One, Austria) and centrifuged at 100*g* for 10 min. The plasma in the top layer was transferred into new tubes and centrifuged at 400*g* for 10 min. The upper two-third portion of centrifuged plasma was removed, and the remaining one-third resuspended. This suspension is considered as platelet-rich plasma. Activation of PRP was done by adding 200 µL of 10% CaCl_2_ to 5 mL of PRP.

### 2.13 Treatment of macrophage polarization with PRP

IFNγ and LPS-, IL-4 and IL-13, and M-CSF-treated MDMs were treated with PRP at a 1:1 dilution in culture medium for 48 h in 5% v/v CO_2_ at 37°C. The phenotypes of treated MDMs were determined *via* flow cytometry.

### 2.14 Treatment of synovial fluid-induced macrophage polarization with PRP

Following MDM differentiation, the culture medium was removed after 24 h of cell resting, and cells were incubated with synovial fluid at 1:8 dilution in culture medium for 48 h in 5% v/v of CO_2_ at 37°C. Synovial fluid was removed, and cells were washed once with 1× PBS. The synovial fluid-treated MDMs were subsequently incubated with PRP at 1:1 dilution for an additional 48 h in 5% v/v of CO_2_ at 37°C. The phenotypes of treated MDMs were determined *via* flow cytometry.

### 2.15 Statistical analysis

For the *ex vivo* study, different percentages of macrophage subpopulations in the IPFPs and synovial tissues, and the log_2_ fold change values of gene expression between four macrophage subpopulations were compared using one-way analysis of variance (one-way ANOVA) followed by Tukey’s multiple-comparisons test. Different macrophage phenotypes between mild and moderate cartilage destruction severity were compared using the Mann–Whitney *U*-test. Pearson’s correlation was used to calculate the relationship of macrophage phenotypes and cartilage destruction severity as well as the relationship of macrophage phenotypes between two types of joint tissues. For the *in vitro* study, a comparison of the percentage of macrophage subpopulations between untreated and treated conditions was determined using two-way ANOVA. Percentage macrophage polarization and percent macrophage subpopulations after treatment were compared using one-way ANOVA, followed by Tukey’s multiple-comparisons test. All data in this entire study will be calculated using GraphPad Prism (version 8) and presented as mean ± SEM. A probability value (*P*-value) of <0.05 will be considered statistically significant.

## 3 Results

### 3.1 Macrophages in IPFPs and synovial tissues of knee OA joints display a predominant CD11c+CD206+ phenotype

**Figure 1:**
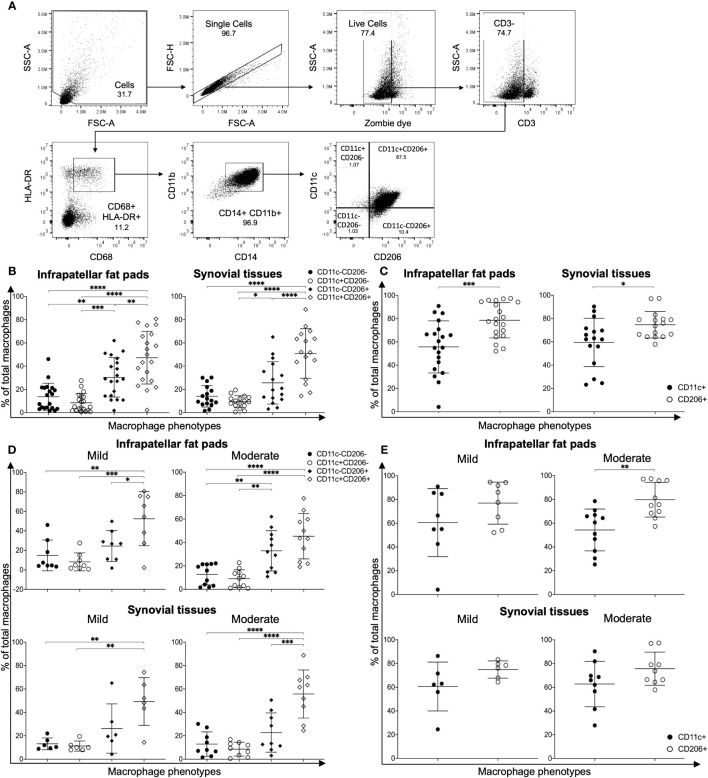
Characterization of macrophage phenotypes in the infrapatellar fat pads (IPFPs) and synovial tissues of patients with knee osteoarthritis (OA). **(A)** Representative gating strategy for the identification of macrophage phenotypes in IPFPs and synovial tissues. Macrophage populations were gated on live CD3-CD68+HLA-DR+CD14+CD11b+ and subsequently gated on CD11C-CD206-, CD11C+CD206-, CD11C-CD206+, and CD11C+CD206+ cells, respectively. **(B, C)** Comparison of macrophage phenotypes in the IPFPs (*n* = 20) and synovial tissues (*n* = 16) obtained from OA patients. **(D, E)** Comparison of macrophage phenotypes in IPFPs and synovial tissues of OA patients with mild (IPFPs; *n* = 8, synovial tissues; *n* = 6) and moderate (IPFPs; *n* = 11, synovial tissues; *n* = 9) OA severity. The differences of CD11c-CD206-, CD11c+CD206-, CD11c-CD206+, and CD11c+CD206+ macrophage frequencies were calculated using one-way ANOVA, and the differences of CD11c+ and CD206+ macrophages were calculated using Mann–Whitney *U*-test analysis (*p < 0.05; **p < 0.01; ***p ≤ 0.001; ****p ≤ 0.0001).

In order to investigate the role of macrophage polarization in OA, macrophages were isolated from IPFPs and synovial tissues of knee OA patients undergoing total knee arthroplasty (TKA). The demographic data of patients whose IPFPs and synovial tissues were obtained for the experiments are shown in **Supplementary Table S2**. The macrophages were identified as CD3-CD68+HLA-DR+CD14+CD11b+ cells (23) and subdivided into CD11c-CD206-, CD11c+CD206- (M1), CD11c-CD206+ (M2), and CD11c+CD206+ macrophages (**Figure 1A**). In both the IPFPs and synovial tissues, the macrophages displayed a predominant CD11c+CD206+ population profile, with the CD11c+CD206+ macrophage percentage being significantly higher than the other populations (**Figure 1B**). In addition, the percentage of CD11c-CD206+ macrophages was also significantly higher than CD11c+CD206- macrophages (**Figure 1B**). A comparison between CD11c and CD206 expression levels between macrophages isolated from IPFPs and synovial tissues showed that there was a significantly higher expression level of CD206 (**Figure 1C**).

Next, we evaluated the correlation between the severity of cartilage destruction with macrophage polarization. The severity of cartilage destruction was determined based on the Mankin score (**Supplementary Table S1**). Our patient population was classified into mild and moderate cases, without any severe cases. In both mild and moderate cases of OA, CD11c+CD206+ macrophages were the predominant population, followed by CD11c-CD206+ macrophages (**Figure 1D**). The profiles between macrophages isolated from IPFPs and synovial tissues and between mild and moderate cases did not differ (**Figure 1D**), nor did the percentages of each macrophage population between mild and moderate OA cases (**Supplementary Figure S1**). The CD206 expression levels were only significantly higher than the CD11c expression levels in macrophages isolated from IPFPs in moderate OA cases, suggesting the role of CD206 expression in OA pathogenesis (**Figure 1E**). The expression levels of CD11c and CD206 also did not differ between mild and moderate OA cases in both IPFPs and synovial tissues (**Supplementary Figure S2**). There was also no significant correlation between Mankin scores and each macrophage population (**Supplementary Figure S3**).

### 3.2 The abundance of macrophages in IPFPs reflects levels in synovial tissues

**Figure 2:**
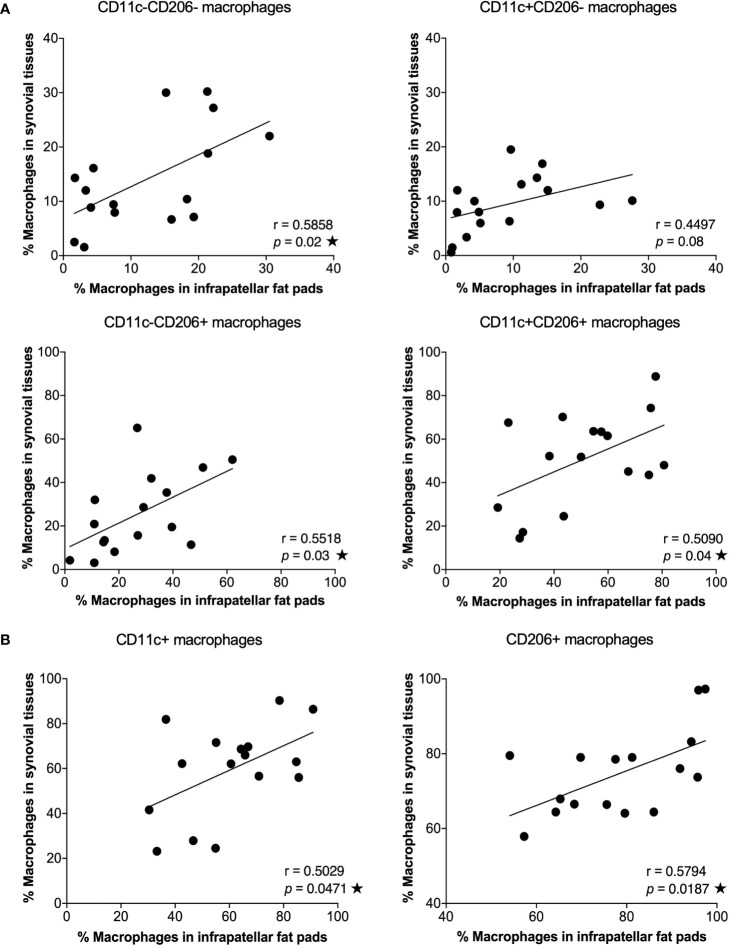
Correlation between macrophage phenotypes in infrapatellar fat pads (IPFPs) and synovial tissues of osteoarthritis (OA) patients. Correlation analysis between the percentages of macrophage phenotypes in IPFPs (x-axis) and synovial tissues (y-axis) of OA patients. **(A)** Correlation of CD11c-CD206-, CD11c+CD206-, CD11c-CD206+, and CD11c+CD206+ macrophages between IPFPs and synovial tissues (*n* = 16). **(B)** Correlation of CD11c+ and CD206+ macrophages between IPFPs and synovial tissues (*n* = 16). Correlations were assessed using Pearson’s correlation coefficient (*r*) test, and any difference with a *p*-value <0.05 was considered statistically significant.

IPFPs and synovial tissues are sites of immune cell infiltration and are implicated in the pathogenesis and pathophysiology of OA (5, 6, 24). However, the microenvironment in these two tissue sites are different (24). The predominant cells of IPFPs are adipocytes, which secrete growth factors, cytokines, and adipokines to sustain IPFP metabolism. In contrast, synovial tissues are mainly composed of fibroblast-like and macrophage-like synoviocytes, which provide an environment for maintaining synovial homeostasis (24). We investigated the distribution of macrophages between IPFPs and synovial tissues in patient-matched samples. The percentages of CD11c-CD206-, CD11c-CD206+, and CD11c+CD206+ macrophages in IPFPs significantly correlated with the macrophage percentages in synovial tissues (*p* = 0.02, *p* = 0.03, and *p* = 0.04, respectively) (**Figure 2A**). In addition, CD11c+ and CD206+ macrophages between IPFPs and synovial tissues also had a significant positive correlation (*p* = 0.0471 and *p* = 0.0187, respectively) (**Figure 2B**). These results show that the macrophages were evenly distributed between the two joint-surrounding tissues.

### 3.3 Gene expression profiles in macrophages isolated from the infrapatellar fat pads of OA patients involved in the inflammatory response, extracellular matrix organization, and osteoclast differentiation

**Figure 3:**
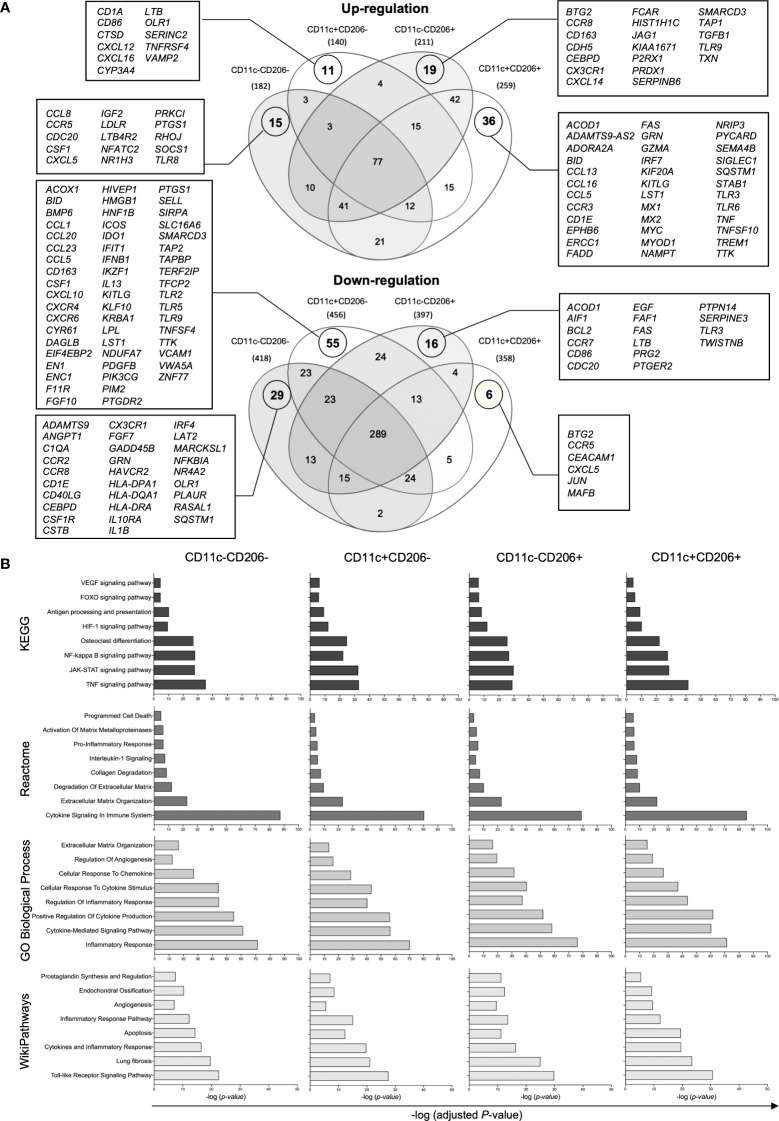
Gene expression analysis of macrophages isolated from the infrapatellar fat pads of osteoarthritis (OA) patients *via* NanoString. **(A)** Venn diagrams demonstrating the overlap of the differentially upregulated and downregulated genes in CD11c-CD206- (*n* = 3), CD11c+CD206- (*n* = 3), CD11c-CD206+ (*n* = 3), and CD11c+CD206+ (*n* = 3) macrophage population compared with internal negative controls. **(B)** The KEGG human pathway 2021, Reactome 2023, GO biological process, and WikiPathways 2021 databases relevant to OA and macrophage function were identified based on differentially expressed genes of CD11c-CD206-, CD11c+CD206-, CD11c-CD206+, and CD11c+CD206+ macrophages. The Y-axis represents the pathway name, and the X-axis represents -log(adjusted *P*-value).

Next, we further investigated the role of each macrophage population by performing a transcriptomic analysis of each macrophage population within IPFPs of knee OA patients. Macrophages were isolated from a pooled sample of IPFPs from 14 knee OA patients per sample. In total, samples from 42 knee OA patients were included in the study. The demographic data of patients whose IPFPs were obtained for the transcriptomic analysis are shown in **Supplementary Table S3**. NanoString analysis was performed on each sorted macrophage subpopulation. From a total of 730 genes, our results show 182, 140, 211, and 259 upregulated genes and 418, 456, 397, and 358 downregulated genes in CD11c-CD206-, CD11c+CD206-, CD11c-CD206+, and CD11c+CD206+ macrophages, respectively (**Figure 3A**). CD11c-CD206-, CD11c+CD206-, CD11c-CD206+, and CD11c+CD206+ macrophages had 15, 11, 19, and 36 uniquely upregulated genes and 29, 55, 16 and six uniquely downregulated genes, respectively (**Figure 3A**). CD11c+CD206+ macrophages had the most number of uniquely upregulated genes and the least number of uniquely downregulated genes (**Figure 3A**). The uniquely upregulated genes expressed in CD11c+CD206+ macrophages are involved in inflammatory responses (*CCL13*, *ADORA2A*, *CCL5*, *SIGLEC1*, *TLR6*, *TNF*, *CCL16*, *CCR3*, and *TLR3*), apoptosis (*PYCARD*, *ADORA2A*, *MYC*, *GZMA*, *MX1*, *FAS*, *FADD*, and *BID*) and TNF signaling (*CCL5*, *FAS*, *FADD*, and *TNF*), whereas the uniquely downregulated genes encode for chemotactic mediators (*CCR5* and *CXCL5*), genes involved in vasculogenesis regulation (*CEACAM1*) and negative regulation of osteoclast differentiation (*MAFB*) (**Figure 3A**). CD11c+CD206- macrophages had the least number of uniquely upregulated genes but the most number of uniquely downregulated genes (**Figure 3A**). The uniquely downregulated genes in CD11c+CD206- macrophages are mainly involved in toll-like receptor signaling pathway (*TLR2*, *TLR5*, and *TLR9*), TNF signaling pathway (*CCL20*, *CCL5*, *CSF1*, *CXCL10*, *IFNB1*, and *VCAM1*), and antigen processing and presentation (*TAP2* and *TAPBP*) (**Figure 3A**).

**Figure 4:**
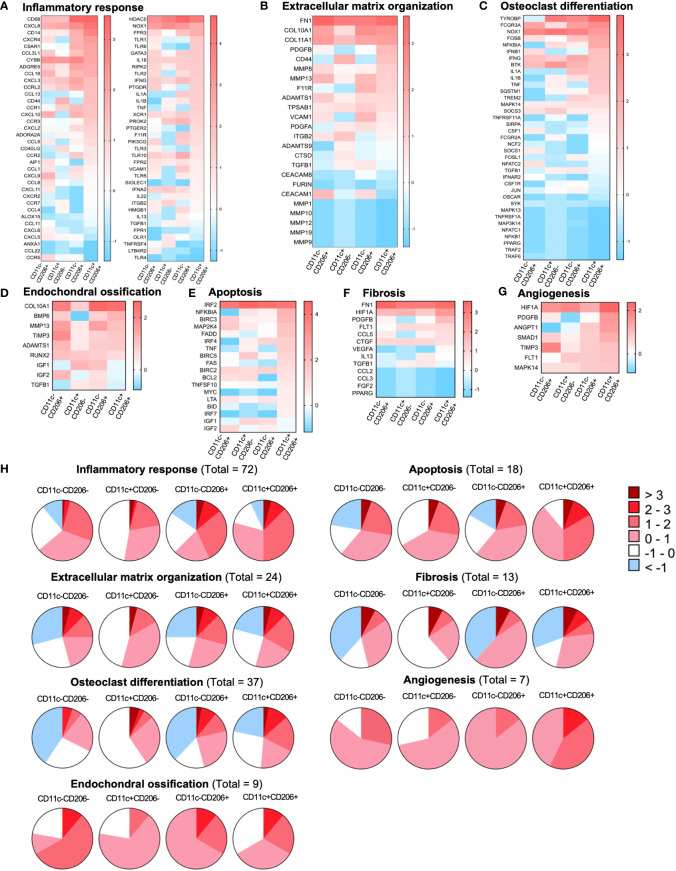
Gene expression profiles of macrophages isolated from the infrapatellar fat pads of osteoarthritis (OA) patients that are involved with OA pathogenesis. Heatmap illustrating the differential expression of genes associated with **(A)** the inflammatory response (GO:0006954), **(B)** extracellular matrix organization (R-HSA-1474244), **(C)** osteoclast differentiation, **(D)** endochondral ossification, **(E)** apoptosis, **(F)** fibrosis, and **(G)** angiogenesis in CD11c-CD206- (*n* = 3), CD11c+CD206- (*n* = 3), CD11c-CD206+ (*n* = 3), and CD11c+CD206+ (*n* = 3) macrophages as the log_2_ fold change of normalized data. Differential gene expressions are displayed as colors ranging from red to blue. **(H)** Pie chart representation of genes differentially expressed in CD11c-CD206-, CD11c+CD206-, CD11c-CD206+, and CD11c+CD206+ macrophages according to inflammatory response, extracellular matrix organization, osteoclast differentiation, endochondral ossification, apoptosis, fibrosis, and angiogenesis pathways.

Differentially expressed genes (DEGs) in CD11c-CD206-, CD11c+CD206-, CD11c-CD206+, and CD11c+CD206+ macrophages were analyzed based on four database platforms: the Kyoto Encyclopedia of Genes and Genomes (KEGG) pathway 2021, the Reactome Pathway 2022 database, the Gene Ontology (GO) Biological Process 2023, and Wikipathways 2021 (**Figure 3B**). We selected eight pathways from each database that reflected macrophage function and may be related to OA pathogenesis with the most highly significant p-values (p<0.05) from each database.. Due to the overlapping genes between the pathways generated from each database, the pathways with the most allocated genes were selected for further analysis. Therefore, the inflammatory responses pathway (GO:0006954), extracellular matrix organization (R-HSA-1474244), osteoclast differentiation pathway, endochondral ossification, apoptosis, fibrosis, and angiogenesis were selected (**Figure 3B**). Heatmaps of DEGs of the selected pathways were generated, and genes were listed based on the ranking of gene expression levels in the CD11c+CD206+ macrophage population (**Figures 4A–G**). When we classified the genes based on their expression levels into <-1, from -1 to 0, 0–1, 1–2, 2–3, and >3 for each macrophage subpopulation and also for each individual pathways, we found that mixed M1/M2 macrophages had the highest number of genes that were upregulated (including the expression levels from 0 to >3) in the inflammatory response, ECM organization, osteoclast differentiation, apoptosis, fibrosis, and angiogenesis (**Figure 4H**). In addition, CD11c-CD206+ macrophages also had the highest number of genes that were upregulated in the endochondral ossification pathways, fibrosis, and angiogenesis (**Figure 4H**).

Due to the high abundance of CD11c+CD206+ macrophages in IPFPs, we selected a number of genes whose expression in CD11c+CD206+ macrophages was high but low in CD11c+CD206- macrophages for further selected analysis. Our results identified two genes, FAS and PDGFB, having significant differences in expression levels among the macrophage populations (**Supplementary Figure S4**). FAS expression in CD11c+CD206+ macrophages was significantly higher than CD11c-CD206+ macrophages, and PDGFB expression in CD11c+CD206+ macrophages was significantly higher than CD11c+CD206- macrophages (**Figure 4H**).

Next, we confirmed the highly inflammatory state of CD11c+CD206+ macrophages and its likelihood in differentiating into osteoclasts (despite the low expression levels of NFATC1 in all macrophage subpopulations; **Supplementary Figure S6A**) by evaluating proinflammatory and anti-inflammatory cytokine production and the expression of TRAP, nuclear factor of activated T-cells, cytoplasmic 1 (NFATC1), matrix metallopeptidase (MMP)-9, and MMP-13 in each macrophage subpopulation. We sorted for CD11c-CD206-, CD11c+CD206-, CD11c-CD206+, and CD11c+CD206+ macrophages and cultured the cells for 24 h. Then, supernatant was collected and examined for the expression levels of TNF-α, IL-1β, IL-6, IL-17A, IL-4, IL-13, IL-10, and IL-1RA *via* cytometric bead array. Our results show that there were no significant differences in the expression levels among the four different macrophage subpopulations (**Supplementary Figure S5**). However, the expression of IL-1β, TNF-α, and IL-6, respectively, in CD11c-CD206+ and CD11c+CD206+ macrophages were above the negative background level and showed an increasing trend, with CD11c+CD206+ expressing these cytokines at the highest levels, followed by CD11c-CD206+ macrophages (**Supplementary Figure S5**). Furthermore, supernatant cultured from sorted CD11c-CD206-, CD11c+CD206-, CD11c-CD206+, and CD11c+CD206+ macrophages were also examined for their MMP-9 and MMP-13 expression *via* ELISA. CD11c+CD206+ macrophages expressed the highest level of MMP-9 and MMP-13 (**Supplementary Figure S6B**). We also performed a flow cytometric analysis evaluating the expression levels of NFATC1 and TRAP in the four macrophage subpopulations. Our results show that CD11c+CD206-, CD11c-CD206+, and CD11c+CD206+ macrophages all had a substantial percentage of NFATC1+TRAP-, which were comparable to one another (**Supplementary Figure S6C**). CD11c+CD206+ macrophages had the highest percentage of NFATC1+TRAP+ cells, of which the percentage was significantly higher than the percentage of NFATC1+TRAP+ cells in CD11c+CD206- and CD11c-CD206- macrophage subpopulations (**Supplementary Figure S6C**). These results suggest a bias in phenotype of CD11c+CD206+ macrophages toward driving inflammation and generating osteoclasts.

### 3.4 Synovial fluid and IPFP- and synovial tissue-conditioned media of knee OA patients induce peripheral blood monocyte-derived macrophage polarization into a phenotype comparable to the CD11c+CD206+ population

**Figure 5:**
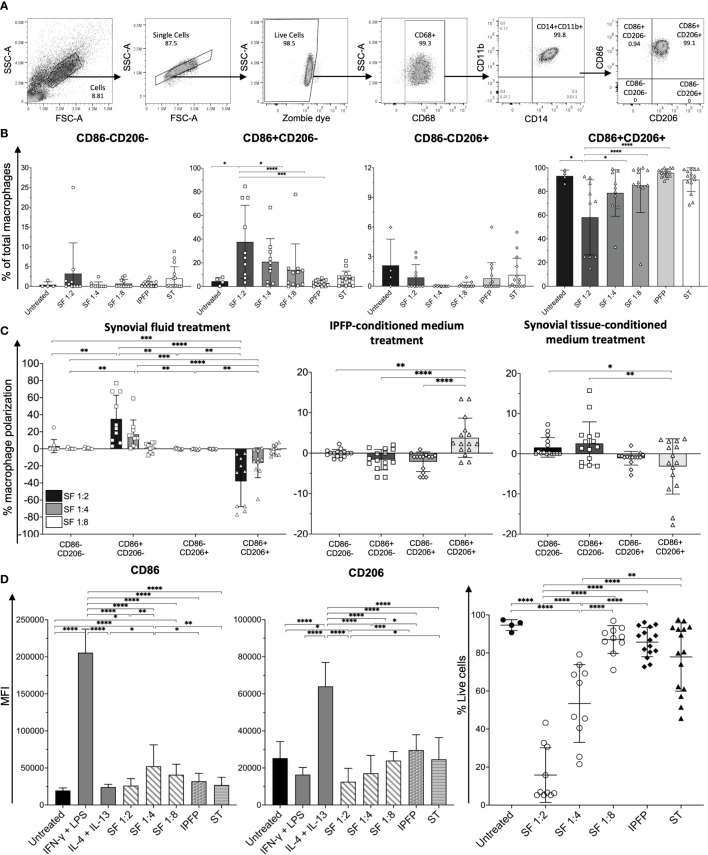
Macrophage profiling of monocyte-derived macrophages (MDMs) treated with synovial fluid and infrapatellar fat pad (IPFP)- and synovial tissue-conditioned media. **(A)** Representative gating strategy for the identification of macrophage phenotypes. Macrophage populations were gated on live CD68+CD14+CD11b+ and subsequently gated on CD86-CD206-, CD86+CD206-, CD86-CD206+, and CD86+CD206+. **(B)** Frequencies, **(C)** polarization status, **(D)** mean fluorescent intensity (MFI) levels of CD86 and CD206, and **(E)** cell viability of CD86-CD206-, CD86+CD206-, CD86-CD206+, and CD86+CD206+ MDMs after treatment with synovial fluid (*n* = 10) at 1:2, 1:4, and 1:8 dilutions and IPFP-conditioned media (*n* = 15) or synovial tissue-conditioned media (*n* = 15) for 48 (h) The differences of CD86-CD206-, CD86+CD206-, CD86-CD206+, and CD86+CD206+ MDM frequencies, polarization status, MFI, and cell viability were calculated using one-way ANOVA (*p < 0.05; **p < 0.01; ***p ≤ 0.001; ****p ≤ 0.0001).

Macrophages are distributed within joint-surrounding tissues, which include synovial fluids, synovial tissues, and IPFPs. To investigate factors that contribute to macrophage polarization in OA, we treated human peripheral blood monocyte-derived macrophages (MDMs) with synovial fluid obtained from knee OA patients at various conditions (1:2, 1:4, and 1:8 dilutions), IPFP-conditioned media, and synovial tissue-conditioned media and determined the changes in the percentages of each macrophage subpopulation. MDMs were identified as CD68+CD14+CD11b+ cells and subcategorized into CD86-CD206-, CD86+CD206-, CD86-CD206+, and CD86+CD206+ macrophages comparable to CD11c-CD206-, “M1” (CD11c+CD206-), “M2” (CD11c-CD206+), and CD11c+CD206+ tissue-resident macrophages, respectively (**Figure 5A**). Each macrophage population was compared among the different treatment conditions. MDMs treated with synovial fluid at 1:2 dilution had significantly higher percentages of CD86+CD206- macrophages than untreated MDMs and MDMs treated with IPFP-conditioned media and synovial fluid at other dilutions (1:4 and 1:8) (**Figure 5B**). In addition, CD86+CD206+ macrophages were significantly lower than untreated MDMs and MDMs treated with IPFP-conditioned media and synovial fluid at other dilutions (1:4 and 1:8) (**Figure 5B**). The stepwise significant decrease of percentages of CD86+CD206- macrophages and significant increase of percentages of CD86+CD206+ macrophages observed demonstrate the effects of synovial fluid on macrophage polarization in a dose-dependent manner (**Figure 5C**). The percentage of macrophage polarization in IPFP-conditioned media-treated MDMs resulted in an increase in CD86+CD206+ macrophage polarization and decreases in CD86+CD206- and CD86-CD206+ macrophage polarization (**Figure 5C**). This was in contrast with MDMs treated with synovial tissue-conditioned media where a decrease in CD86+CD206+ macrophage polarization and an increase in CD86+CD206- macrophage polarization were observed (**Figure 5C**).

Despite the synovial fluid at 1:2 dilution resulting in the highest percentage of CD86+CD206- macrophages and a decrease in CD86+CD206+ macrophages, MDMs in this condition had only 20%–30% cell viability (**Figure 5E**). Interestingly, MDMs treated with synovial fluid at 1:8 dilution had >80% cell viability, which had similar levels of cell viability to MDMs treated with IPFP-conditioned media and synovial tissue-conditioned media (**Figure 5E**). MDMs treated with synovial fluid at 1:8 dilution still had significant increases of CD86 when compared to untreated MDMs (**Figure 5D**), suggesting the effects of synovial fluid upon macrophage polarization. These results demonstrate the different microenvironment in driving macrophage polarization in joint-surrounding tissues. Moreover, synovial fluid and factors within synovial tissues may also partially contribute to macrophage cell death to some extent.

### 3.5 Increased proportions of CD86+CD206+ macrophages from synovial fluid treatment can be decreased with platelet-rich plasma treatment

**Figure 6:**
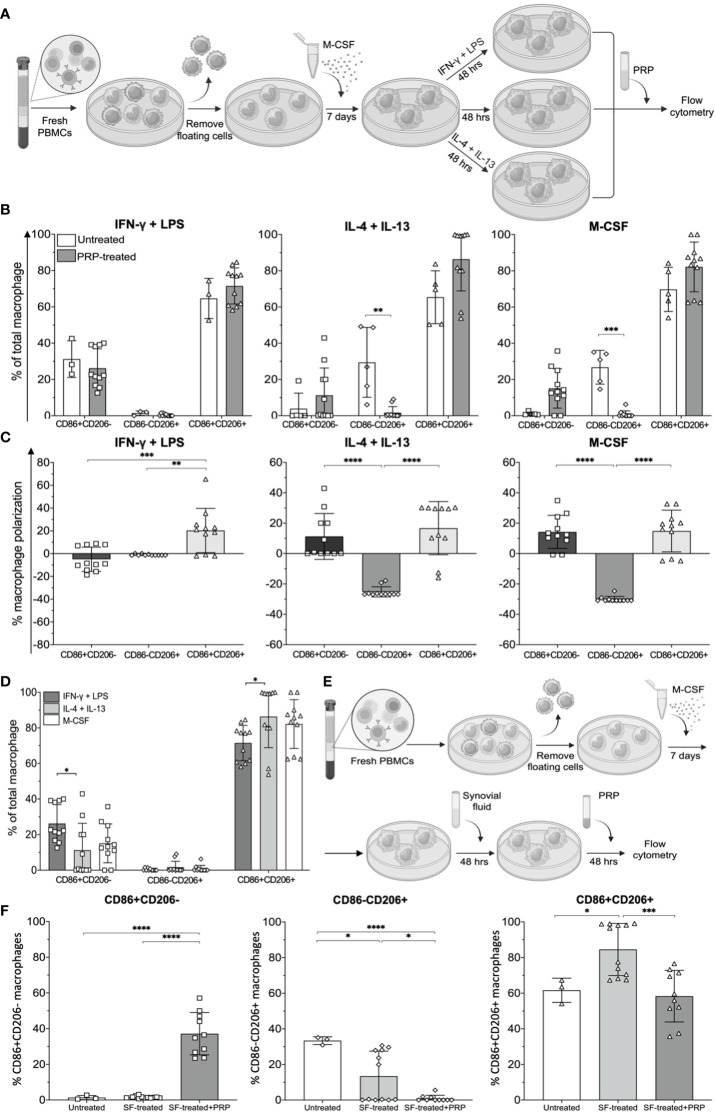
Effects of platelet-rich plasma (PRP) on synovial fluid-treated monocyte-derived macrophages (MDMs). **(A)** Schematic of MDM polarization simulating CD11c+CD206- (M1), CD11c-CD206+ (M2), and CD11c+CD206+ macrophages using IFNγ and LPS, IL-4 and IL-13, and M-CSF, respectively, and subsequent treatment with PRP. **(B)** Frequencies and **(C)** polarization status of MDMs after treatment with PRP at a 1:8 dilution (*n* = 11). **(D)** Comparison of macrophage subpopulation frequencies after treatment with PRP (*n* = 11). **(E)** Schematic illustrating MDMs receiving treatment with PRP after being exposed to synovial fluid. **(F)** Comparison of CD86+CD206-, CD86-CD206+, and CD86+CD206+ macrophage frequencies after treatment with synovial fluid only (*n* = 12) and synovial fluid followed by PRP (*n* = 10). The differences of CD86-CD206-, CD86+CD206-, CD86-CD206+, and CD86+CD206+ MDM frequencies, polarization status, mean fluorescent intensity, and cell viability were calculated using one-way ANOVA (*p < 0.05; **p < 0.01; ***p ≤ 0.001; ****p ≤ 0.0001).

Platelet-rich plasma is an autologous concentrate of platelets in a small volume of plasma and has been used to relieve pain in OA (25). Therefore, we tested the effects PRP may have on macrophage polarization. Peripheral blood monocytes were treated with M-CSF, IFNγ and LPS, or IL-4 and IL-13 to simulate CD11c+CD206+, CD11C+CD206- (M1), and CD11c-CD206+ (M2) macrophage phenotype *in vitro* models, respectively. Then, these cells were treated with PRP for 48 h (**Figure 6A**). The treatment of the three macrophage populations resulted in significant decreases in CD86-CD206+ macrophages in IL-4 and IL-13-treated and M-CSF-treated conditions (**Figure 6B**). In addition, in all three conditions of macrophages, CD86-CD206+ macrophages all decreased to nearly baseline levels (**Figure 6B**). We also calculated the changes in percentage of macrophage polarization to demonstrate the increase or decrease in each macrophage subpopulation for all culture conditions. PRP treatment resulted in an increase in CD86+CD206+ macrophage popularization in all culture conditions (IFNγ and LPS, IL-4 and IL-13, and M-CSF treatment), and these increases were significantly higher than the polarizations of other macrophage subsets for IFNγ and LPS-treated macrophages and significantly higher than the polarizations of CD86-CD206+ macrophages in IL-4 and IL-13-treated macrophages and M-CSF-treated macrophages (**Figure 6C**). In two out of the three conditions, there was a decrease in CD86-CD206+ (M2) macrophage polarization, suggesting a reduction in the percentage of this population (**Figure 6C**). Similarly, the effects of PRP on CD86+CD206- macrophages showed an increase of this macrophage subpopulation in two out of the three conditions tested. In IFNγ and LPS-treated macrophages, there was only a slight decrease in polarization, whereas in IL-4 and IL-13-treated macrophages and M-CSF-treated macrophages, there was a 10%–20% reduction in polarization (**Figure 6C**). Despite the slight difference in macrophage polarization among the three macrophage conditions, the resulting profiles of macrophages were similar to one another in that CD86+CD206+ macrophages remained predominant (mean levels of 80%), followed by CD86+CD206- and CD86-CD206+ macrophages, respectively (**Figure 6D**). However, there were still significant differences between the percentages of CD86+CD206- and CD86+CD206+ macrophage populations in the IFNγ and LPS-treated condition and IL-4 and IL-13-treated condition (**Figure 6D**).

Next, MDMs differentiated by M-CSF treatment were treated with synovial fluid at 1:8 dilution prior to treatment with PRP for another 48 h (**Figure 6E**). Our results show that synovial fluid treatment significantly increased the percentage of CD86+CD206+ macrophages, but treatment with PRP restored the percentage of CD86+CD206+ macrophages to similar levels as those of untreated MDMs (**Figure 6F**). After treatment with PRP, the percentages of CD86+CD206- macrophages also significantly increased, but the percentages of CD86-CD206+ macrophages significantly decreased (**Figure 6F**). Therefore, the new profiling of macrophages after PRP treatment was a lower proportion of CD86+CD206+ (yet still predominant), followed by CD86+CD206- and CD86-CD206+ macrophages, respectively.

## 4 Discussion

Macrophages are predominant immune cells that infiltrate IPFPs and synovial tissues of OA patients (7–11, 26, 27). They are highly plastic cells that undergo polarization in response to altered environmental stimuli or pathological conditions (11, 15). Many diseases describe macrophages in terms of their M1 vs. M2 polarization. M1 and M2 macrophages are identified based on their cell surface expression of certain surrogate CD markers (CD11c+CD206- and CD11c-CD206+, respectively) and their gene expression profiling. In our study, we show that macrophages in IPFPs and synovial tissues of knee OA patients display CD11c+CD206+, followed by CD206+CD11c- (M2), CD11c-CD206-, and CD11C+CD206- (M1) macrophages, respectively. The macrophage subset profiling was similar between mild and moderate cases of OA. However, only in IPFPs of moderate cases of knee OA was CD206 expression significantly higher than CD11c expression. Nonetheless, the abundance of CD11c-CD206-, CD11c-CD206+, CD11c+CD206+, CD11c+, and CD206+ macrophages between the two tissues was significantly correlated. CD11c+CD206+ macrophages in adipose tissues are due to have a higher inflammatory cytokine expression than single positives (CD11c+CD206- macrophages and CD11c-CD206+ macrophages) (28). Adipose tissue is known to regulate osteoarthritis as lipodystrophic mice do not develop spontaneous OA nor injury-induced OA despite receiving high fat diet (29). IPFPs are adipose tissues with closest proximities to the joint cavity (30) and therefore were the focus of this study.

A transcriptomic analysis of CD11c-CD206-, CD11c+CD206-, CD11c-CD206+, and CD11c+CD206+ macrophages from IPFPs of knee OA patients identified a number of both shared genes and uniquely expressed genes among the macrophage subpopulations. A number of pathways related to OA pathogenesis were investigated, whereby CD11c+CD206+ macrophages had the most upregulated genes that were involved in inflammatory responses, ECM organization, osteoclast differentiation, apoptosis, fibrosis, and angiogenesis, while CD11c-CD206+ macrophages had the most upregulated genes involved in endochondral ossification, fibrosis, and angiogenesis. CD11c+CD206+ macrophages did indeed express the highest levels of IL-1β, IL-6, TNF-α, MMP-9, and MMP-13 and had the highest proportion of NFATC1+TRAP+ cells, which signifies osteoclast differentiation. The inflammatory response generates cytokines (IL-1β and TNFα) that further induce IL-6, IL-8, MCP-1, and MMP production from synovial fibroblasts and chondrocytes (10, 33, 34). IL-6, IL-8, and MCP-1 are known for their induction of inflammation and leukocyte recruitment (35). MMPs are cartilage-degrading enzymes, of which macrophages can secrete MMPs-1, -2, -3, -8, -9, -11, -12, -13, and -14 (36). IL-1β and TNFα also directly induces osteoclast precursors to differentiate into mature osteoclasts (37). COL10A1, a gene from the ECM organization group that was expressed highly in CD11c+CD206+ macrophages, may affect the deposition of other matrix molecules in articular cartilage, providing a proper environment for hematopoiesis and mineralization and promoting endochondral ossification (38). Osteoclasts are bone-resorbing cells and are recruited by RANKL during post-traumatic OA (39). IPFP-isolated CD11c+CD206+ macrophages expressed genes related to the RANK signaling pathway, reflecting the possible ability for macrophages to differentiate into osteoclasts, which may lead to increased osteoclastogenesis (40). Synovial fluid macrophages exposed to M-CSF and RANKL were able to differentiate into osteoclast-like cells (41). Osteoclast-mediated bone resorption also causes subchondral bone remodeling (42). The discrepancy between fibrosis due to an attempt to rebuild ECM in OA can lead to pain and stiffness of the joints (43). Fibrosis is observed in IPFPs of experimental mouse models and OA patients (44, 45). In knee OA rat models, inhibition of synovial macrophage pyroptosis reduces fibrosis (46). Angiogenesis is also implicated in OA symptomatology as new blood vessels contribute to joint inflammation and pain (47, 48). CD11c+CD206+ and CD11c-CD206+ macrophage populations comprised the majority of macrophages in IPFPs and expressed genes that were directly relevant to OA pathogenesis as mentioned above. Combining these two factors results in IPFP-infiltrating macrophages that are prone to drive OA pathology and enacts IPFP-residing macrophages as key players in driving OA pathogenesis.

Despite observing TRAP+NFATC1+ macrophages in the different macrophage subpopulations up to approximately 50%–60% of CD11c+CD206+ macrophages, the gene expression levels of NFATC1 and MMP13 were low in all four macrophage subpopulations. This may be due to macrophages being in an early state of osteoclastogenesis as NFATC1 is a regulator for the terminal differentiation of human osteoclasts (49, 50) or the fact that the metabolic environment of the host sustains these macrophages into a state of being an osteoclast precursor (51).

The number of uniquely expressing genes of CD11c+CD206+ macrophages was the highest, and CD11c+CD206- macrophages were the lowest among the macrophage populations. Genes that were highly expressed in CD11c+CD206+ macrophages but expressed at low levels or downregulated in CD11c+CD206- macrophages were selected. FAS and PDGFB expression levels in CD11c+CD206+ macrophages were two genes that had significantly higher expression levels than when expressed in CD11c-CD206+ and CD11c+CD206- macrophages, respectively. FAS encodes for the Fas cell death receptor and function by engagement with its ligand (FasL) (52). Fas-associated protein with DD (FADD) adaptor proteins are recruited, and caspase-8 and caspase-10 are activated, resulting in the induction of cell apoptosis (52). In a rheumatoid arthritis model, Fas receptors on macrophage also resulted in the formation of FADD containing DISCs (53). Higher susceptibility to FasL (Fas ligand)-induced apoptosis was observed in tenocytes of OA patients (54). Platelet-derived growth factor (PDGF)-BB was demonstrated to have a role in subchondral bone angiogenesis in a DMM mice model (55).

Further understanding of joint tissue-residing macrophage polarization in OA is exemplified by understanding of the microenvironment which induces the macrophages. Synovial fluid-treated MDMs resulted in a significant increase in CD86+CD206- macrophages in a dose-dependent manner. This was different to IPFP- and synovial tissue-conditioned media where there were no significant changes in the proportion of macrophage subsets. Despite synovial fluid-treated MDMs having higher proportions of CD86+CD206- macrophages when MDMs were treated with higher concentrations of synovial fluid, there was also a higher level of significant cell death. This may be due to the significantly higher expression of Fas observed in CD11c+CD206+ macrophages and FADD as a uniquely expressing gene in the CD11c+CD206+ macrophage population from the transcriptomic analysis. However, FADD itself has numerous functions, which include cell death, proliferation, innate immunity, and inflammation (56). Nonetheless, synovial fluid at the lowest concentration tested in this study (1:8 dilution) still influenced the outcome of macrophage polarization profiling as seen with significant increases in both CD86 and CD206, suggesting the actual effects of mediators within the synovial fluid in driving macrophage polarization. When MDMs were cultured with IPFP-conditioned media, the originally high levels of CD86+CD206+ macrophages still underwent a further significant expansion of the population. However, in synovial tissue-conditioned media-treated MDMs, there was a reduction in the CD86+CD206+ macrophage population and a rather significant increase in the CD86+CD206- macrophage population. These results demonstrate the different milieus between IPFPs, synovial tissues, and synovial fluid that partake in driving macrophage polarization.

Lastly, in this study, we demonstrate that PRP have features to shift macrophage polarization in favor of CD86+CD206- macrophages, representative of M1 (CD11c+CD206-) macrophages. PRP treatment of synovial fluid-treated MDMs resulted in changes in macrophage subset profiling into a predominant CD86+CD206+, followed by CD86+CD206- macrophages and with very low levels of CD86-CD206+ macrophages. However, a slight discrepancy was observed between synovial fluid-treated MDMs in the comparison studies with other conditioned media and synovial fluid-treated MDMs in the PRP experiments in that the final macrophage profiling was different. This may be due to the timing of *in vitro* culturing and treatment of the cells. In the conditioned media comparison experiments, synovial fluid-treated MDMs had a lower time exposure to synovial fluid. The longer exposure time between synovial fluid and MDMs may have caused increased unnecessary cell death that was observed when synovial fluid was treated with MDMs at a higher concentration.

Despite our best efforts, there still remains many limitations that prohibit accurate and deep understanding of macrophages in joint-surrounding tissues of OA from this study. These limitations include a lack of joint tissue samples from healthy individuals due to ethical reasons, a limited number of tissue samples, the low number of macrophages isolated from each tissue of a given patient, and the low yield of macrophage RNA extraction due to the nature of the cells themselves. For these reasons, our study included datasets from a NanoString technology, which required the least number of RNA content. In addition, macrophage heterogeneity and their substantial plasticity in tissues would warrant for further single cell analysis, *in vivo* studies, and cell barcoding for proper classification of macrophages and their function in these tissues. PRP treatment in this study was also generated from the peripheral blood of healthy individuals. However, in the clinical setting, an autologous PRP is generated from the peripheral blood of OA patients themselves, which will most likely have systemic inflammation to some extent (57). Further follow-up studies may include testing of the inflammatory mediators at a broader extension to identify the responsible factor that is generated from macrophages.

In conclusion, our study provides an insight into the key characteristics of macrophages isolated from adipose tissues that underlie the patella in knee OA. The features of these macrophages are linked to key features that are found in OA pathology. In addition, we have also demonstrated shifting macrophage profiling *in vitro* with a treatment modality used in the clinics that can be personalized to fit each OA patient.

## Data availability statement

The data presented in the study are deposited in the GEO repository, accession number GSE252542.

## Ethics statement

The studies involving humans were approved by Institutional Review Board (IRB) at the Faculty of Medicine, Chulalongkorn University (0734/65). The studies were conducted in accordance with the local legislation and institutional requirements. The participants provided their written informed consent to participate in this study.

## Author contributions

PH: Data curation, Formal analysis, Methodology, Writing – original draft. NL: Data curation, Investigation, Methodology, Writing – review & editing. PS: Formal analysis, Methodology, Resources, Supervision, Validation, Writing – review & editing. JW: Data curation, Methodology, Resources, Writing – review & editing. TC: Data curation, Methodology, Resources, Writing – review & editing. MT: Investigation, Methodology, Project administration, Resources, Supervision, Writing – review & editing. SN: Methodology, Resources, Writing – review & editing. AT: Investigation, Methodology, Project administration, Resources, Supervision, Writing – review & editing. TP: Conceptualization, Investigation, Methodology, Resources, Supervision, Writing – review & editing. RR: Conceptualization, Formal analysis, Funding acquisition, Methodology, Project administration, Resources, Supervision, Validation, Visualization, Writing – original draft, Writing – review & editing.
