# Supplementary material for: Infrapatellar fat pad adipose tissue-derived macrophages display a predominant CD11c+CD206+ phenotype and express genotypes attributable to key features of OA pathogenesis
Source: Front Immunol. 2024 Feb 1;15:1326953. doi: 10.3389/fimmu.2024.1326953 (PMC10867170; doi:10.3389/fimmu.2024.1326953)
Supplement: Supplementary file 1 [file DataSheet_1.pdf]

Supplementary Material

1 Supplementary Figures

1.1 Figure S1

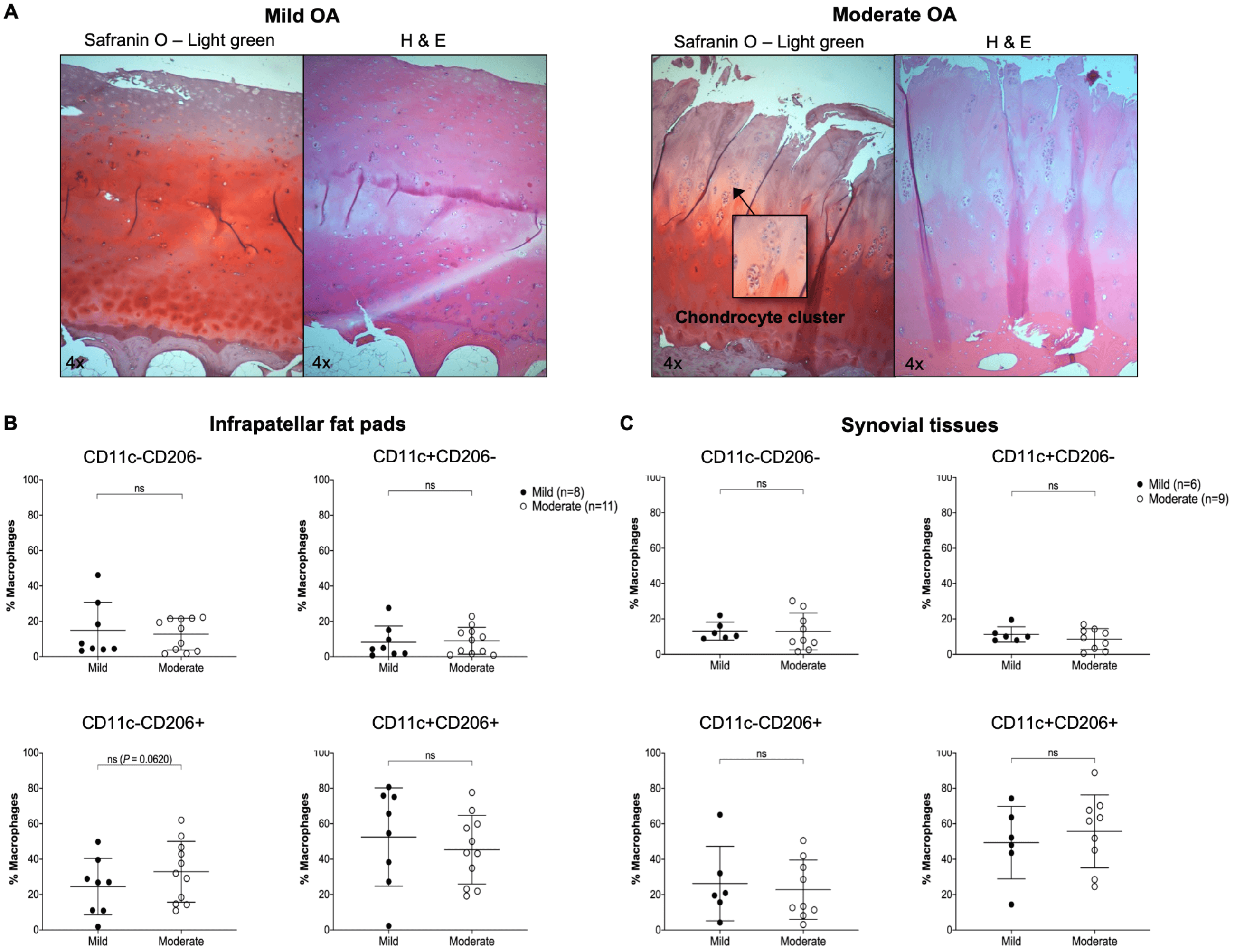

Supplementary Figure S1: Comparison of CD11c-CD206-, CD11c+CD206-, CD11c-CD206+ and CD11c+CD206+ macrophage phenotype frequencies in IPFPs and synovial tissues of knee OA patients according to the severity of cartilage destruction. (A) Representative histological staining of articular cartilage of knee OA patients with mild and moderate cartilage destruction. Safranin-O & light green and H&E stain; picture taken with 4x objectives. (**B**) The frequencies of CD11c-CD206-, CD11c+CD206-, CD11c-CD206+ and CD11c+CD206+ macrophage phenotypes in IPFPs were compared between patients with mild (n = 8) and moderate (n = 11) OA severity. (**C**) The frequencies of CD11c-CD206-, CD11c+CD206-, CD11c-CD206+ and CD11c+CD206+ macrophage phenotypes in synovial tissues were compared between patients with mild (n = 6) and moderate (n = 9) OA severity. The differences of macrophage phenotype frequencies between mild and moderate OA severity were calculated using Mann-Whitney U test analysis (*, $p < 0.05$).

1.2 Figure S2

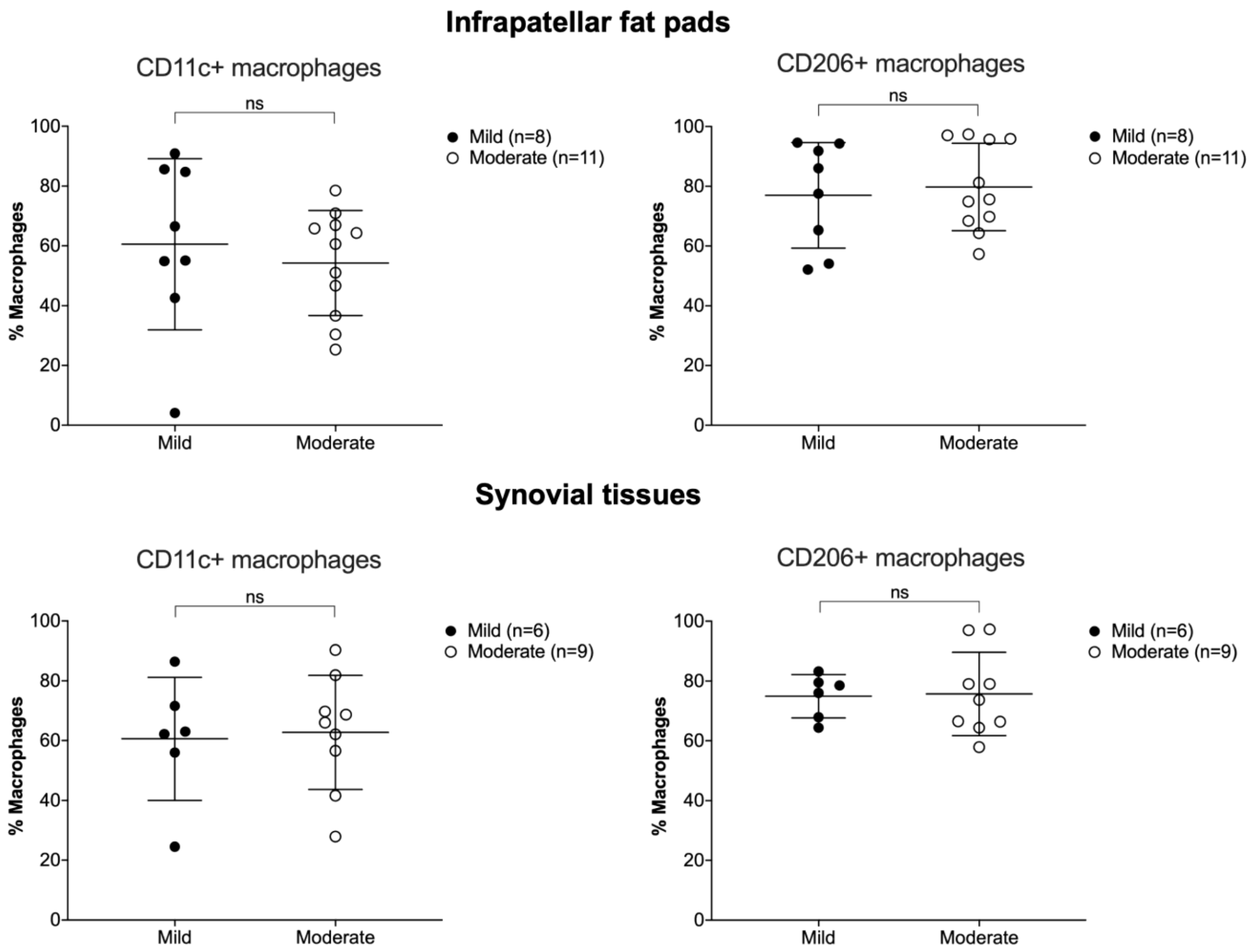

Supplementary Figure S2: Comparison of CD11c+ and CD206+ macrophage phenotypes in the IPFPs and synovial tissues of knee OA patients according to the severity of cartilage destruction.
(A) The frequencies of macrophage phenotypes (CD11c+ and CD206+ macrophages) in the IPFPs were compared between patients with mild (n = 8) and moderate (n = 11) OA severity. (B) The frequencies of macrophage phenotypes (CD11c+ and CD206+ macrophages) in synovial tissues were compared between patients with mild (n = 6) and moderate (n = 9) OA severity. The differences of macrophage phenotype frequencies between mild and moderate OA severity were calculated using Mann-Whitney U test analysis. (*, $p < 0.05$).

1.3 Figure S3

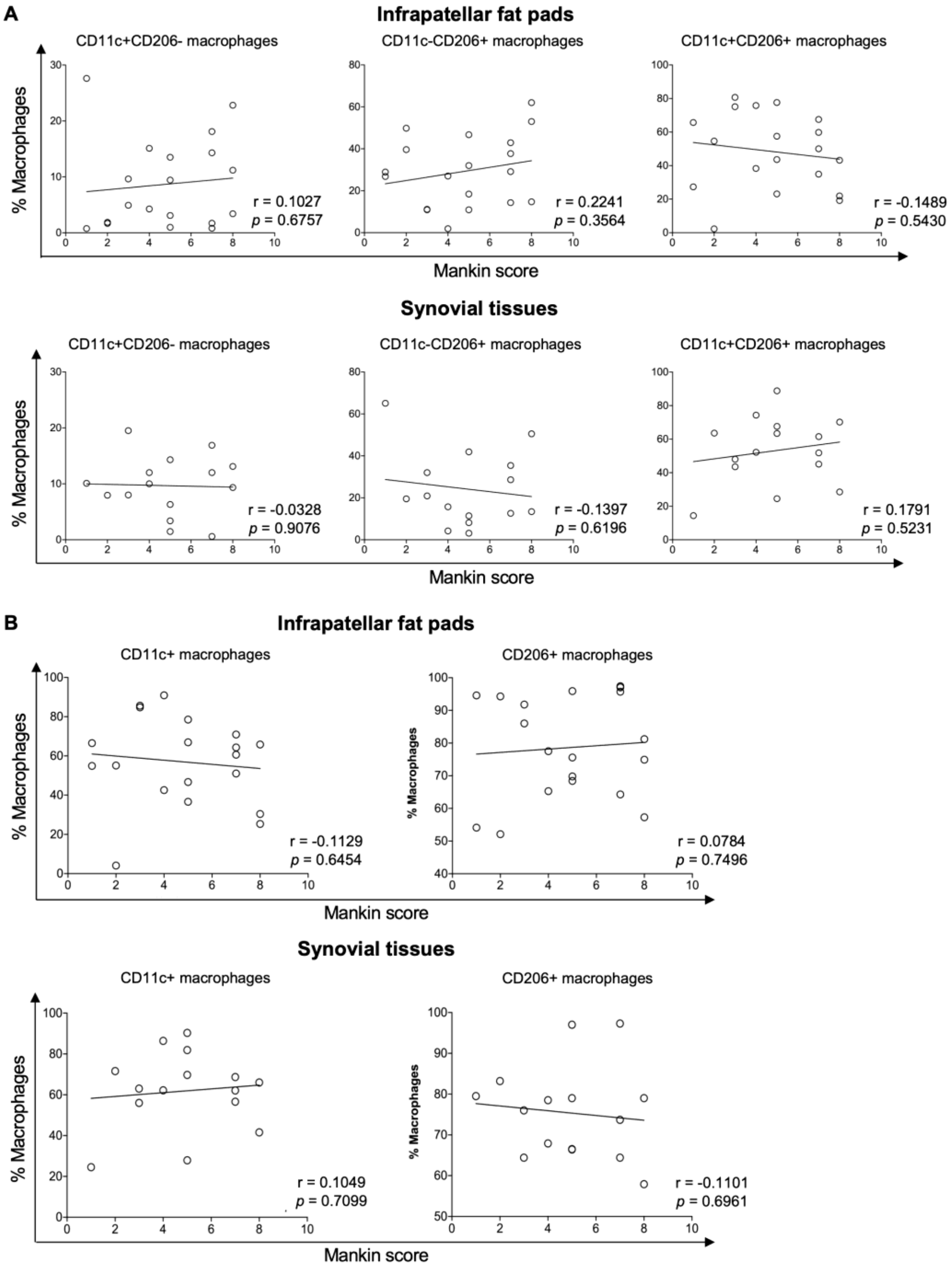

Supplementary Figure S3: Correlation between macrophage phenotypes in IPFPs and synovial tissues of knee OA patients and MANKIN scores. Correlation between CD11c+CD206-, CD11c-CD206+ and CD11c+CD206+ (A) or CD11c+ and CD206+ macrophages (B) with MANKIN scores were evaluated using Pearson's correlation coefficient (r) test. Any differences with a *p*-value of < 0.05 was considered statistically significant.

1.4 Figure S4

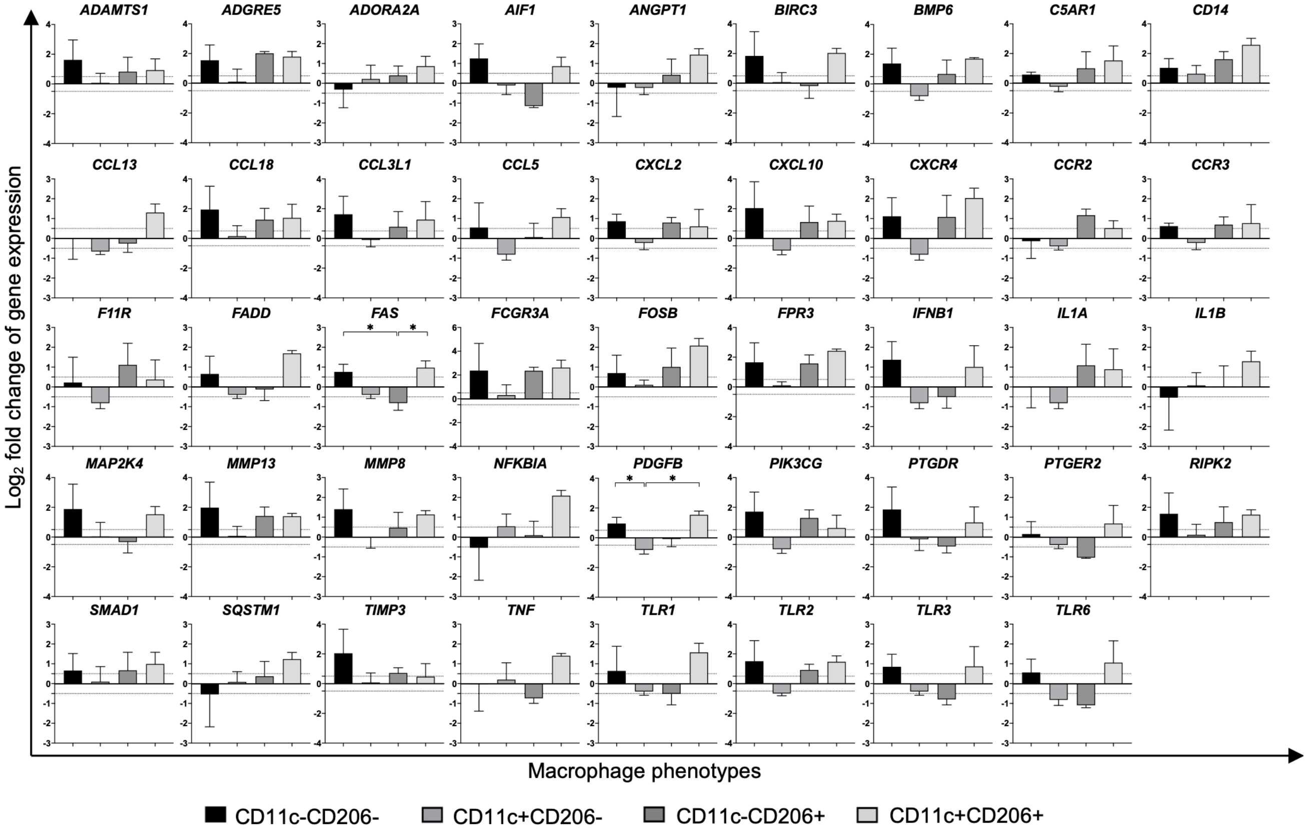

Supplementary Figure S4: Bar graphs comparing gene expression levels of selected genes from the inflammatory response, extracellular matrix organization, osteoclast differentiation, endochondral ossification, apoptosis, fibrosis and angiogenesis pathways among macrophage phenotype populations. The differences in gene expression between CD11c-CD206- (n=3), CD11c+CD206- (n=3), CD11c-CD206+ (n=3) and CD11c+CD206+ (n=3) macrophages were calculated using one-way ANOVA, and any differences with a p-value of 0.05 was considered statistically significant. (*, $p < 0.05$; **, $p < 0.01$).

1.5 Figure S5

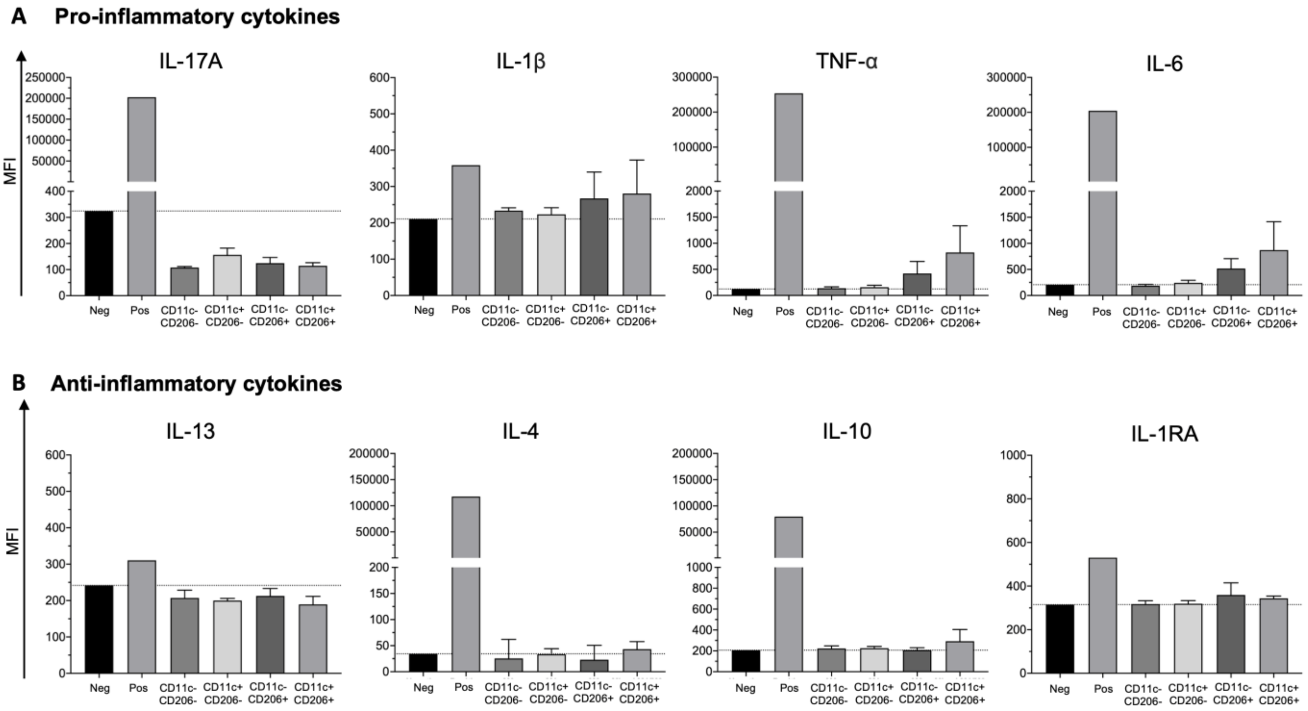

Supplementary Figure S5: Pro-inflammatory and anti-inflammatory mediators from macrophage subpopulations isolated from infrapatellar fat pads of knee OA patients. Bar graphs comparing MFI levels of (A) pro-inflammatory cytokines (IL-17A, IL-1β, TNF-α and IL-6) and (B) anti-inflammatory cytokines (IL-13, IL-4, IL-10 and IL-1RA) among macrophage phenotype populations. The differences in gene expression between CD11c-CD206- (n=3), CD11c+CD206- (n=3), CD11c-CD206+ (n=3) and CD11c+CD206+ (n=3) macrophages were calculated using one-way ANOVA. Any differences with a p-value of 0.05 were considered statistically significant.

1.6 Figure S6

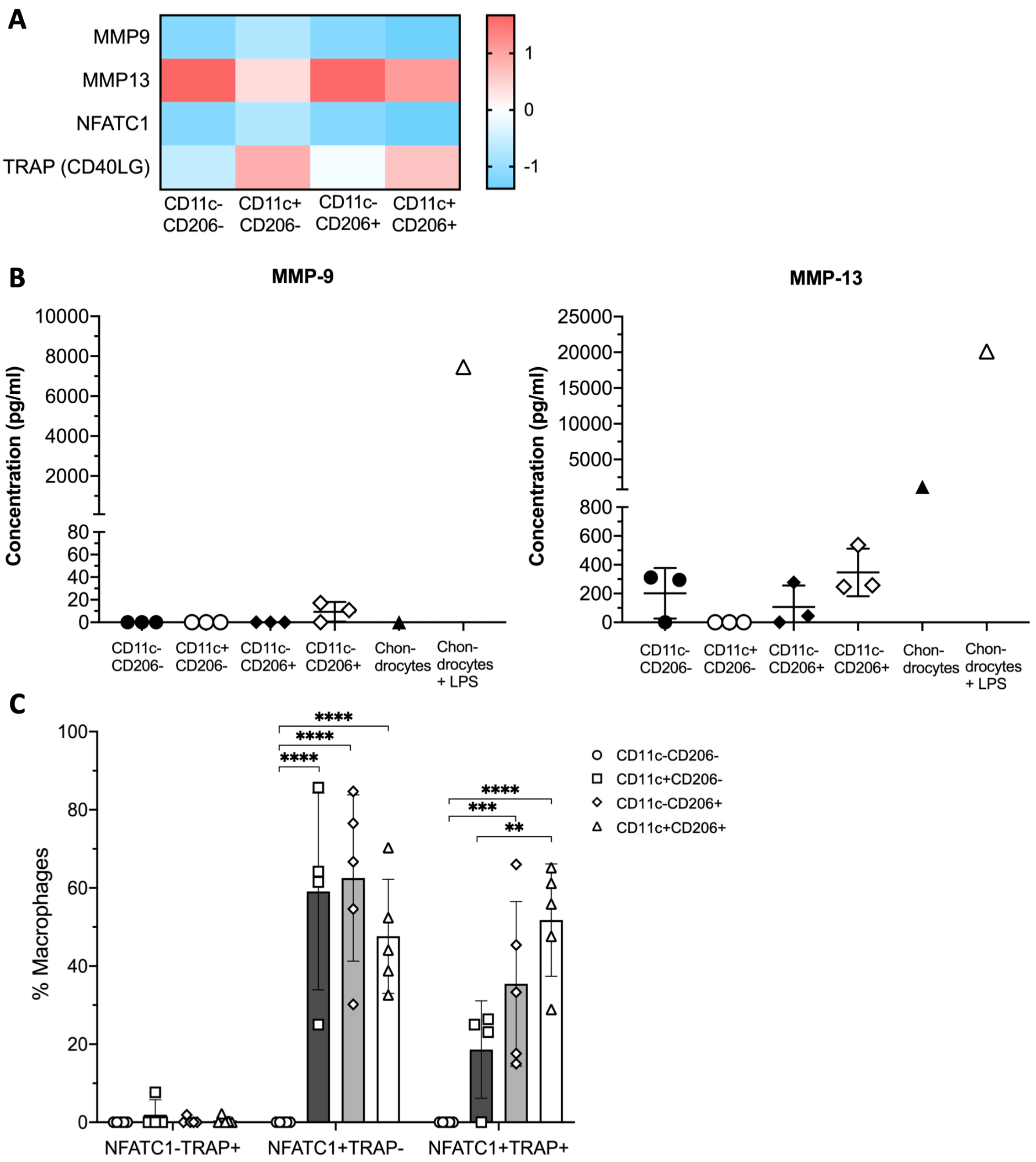

Supplementary Figure S6: Expression of important factors for osteoclastogenesis in macrophage subpopulations isolated from infrapatellar fat pads of knee OA patients. (A) Heatmap illustrating the differential expression of genes in osteoclastogenesis. (B) Comparison of MMP-9 and MMP-13 production levels in CD11c-CD206- (n=3), CD11c+CD206- (n=3), CD11c-CD206+ (n=3) and CD11c+CD206+ (n=3) macrophages. (C) Comparison of NFATc1 and TRAP expression frequencies of four macrophage subpopulations (n=5). The differences in MMP concentrations among macrophage phenotype populations and macrophage frequencies were calculated using one-way ANOVA (*, $p < 0.05$; **, $p < 0.01$; ***, $p \leq 0.001$, ****, $p \leq 0.0001$).

2 Supplementary Tables

2.1 Supplementary Table S1: Mankin scoring system with individual parameters and scores (57).

Feature	Score
**I. Cartilage surface structure**	
A. Normal	0
B. Surface irregularities	1
C. Pannus and surface irregularities	2
D. Clefts to transitional zone	3
E. Clefts to redial zone	4
F. Clefts to calcified zone	5
G. Complete disorganization	6
**II. Cells (arrangement of chondrocytes)**	
A. Normal	0
B. Diffuse hypercellularity	1
C. Chondrocyte cloning or cluster	2
D. Hypocellularity	3
**III. Safranin-O staining (background staining intensity)**	
A. Normal	0
B. Slight reduction	1
C. Moderate reduction	2
D. Severe reduction	3
E. No dye noted	4
**IV. Tidemark integrity**	
A. Intact	0
B. Crossed by blood vessels	1
**Total**	14

2.2 **Supplementary Table S2:** Demographic data of patients whose tissue samples were included in the entire study

Demographic feature	Frequency (n = 101)
**Gender**	
Male	23
Female	78
**Age (years)**	72.8 ± 8.98
< 60	7
60 - 70	30
71 - 80	36
> 90	28
**Weight (kg)**	64.8 ± 13.1
**Hight (cm)**	156 ± 7.92
**Body Mass Index (kg/m^2)**	26.4 ± 4.89
< 18.5	2
18.5 - 24.9	43
25 - 29.9	37
30 - 34.9	14
35 - 39.9	3
> 40	2

2.3 Supplementary Table S3: Demographic data in patients that were used for the Nanostring analysis

Demographic feature	Frequency (n = 42)
**Gender**	
Male	8
Female	34
**Age (years)**	74.0 ± 8.32
< 60	1
60 - 70	13
71 - 80	16
> 90	12
**Weight (kg)**	64.5 ± 11.5
**Hight (cm)**	155 ± 8.19
**Body Mass Index (kg/m^2)**	26.5 ± 4.99
< 18.5	0
18.5 - 24.9	20
25 - 29.9	15
30 - 34.9	5
35 - 39.9	1
> 40	1
